# PARP Inhibitors as Therapeutics: Beyond Modulation of PARylation

**DOI:** 10.3390/cancers12020394

**Published:** 2020-02-08

**Authors:** Ahrum Min, Seock-Ah Im

**Affiliations:** 1 Cancer Research Institute, Seoul National University College of Medicine, Seoul 03080, Korea; mar6716@hanmail.net; 2 Biomedical Research Institute, Seoul National University Hospital, Seoul 03080, Korea; 3 Department of Internal Medicine, Seoul National University Hospital, Seoul 03080, Korea; 4 Translational Medicine, Seoul National University College of Medicine, Seoul 03080, Korea

**Keywords:** PARP, PARP inhibitors, PARylation, trapping, cancer therapeutic strategy

## Abstract

Poly (ADP-ribose) polymerase (PARP) 1 is an essential molecule in DNA damage response by sensing DNA damage and docking DNA repair proteins on the damaged DNA site through a type of posttranslational modification, poly (ADP-Ribosyl)ation (PARylation). PARP inhibitors, which inhibit PARylation through competitively binding to NAD+ binding site of PARP1 and PARP2, have improved clinical benefits for BRCA mutated tumors, leading to their accelerated clinical application. However, the antitumor activities of PARP inhibitors in clinical development are different, due to PARP trapping activity beyond blocking PARylation reactions. In this review, we comprehensively address the current state of knowledge regarding the mechanisms of action of PARP inhibitors. We will also discuss the different effects of PARP inhibitors in combination with cytotoxic chemotherapeutic agents regarding the mechanism of regulating PARylation.

## 1. Introduction

All cells have more than tens of thousands of events that damage DNA in multiple ways, ranging from single base mismatches, bulky adducts in DNA bases, intra- and inter-strand DNA crosslinks, to single- and double-strand breaks (SSBs and DSBs) [1,2]. This DNA damage threatens genomic stability. There are DNA damage responses (DDRs), the sophisticated mechanisms of genome protection in cells, that function to activate the cell cycle checkpoint pathway to maintain genome stability by stopping or delaying the cell cycle during DNA damage or unstable DNA replication to allow the repair of damaged DNA lesions. DDRs activate transcription of a repair molecule or pro-apoptotic molecule to cause overexpression of the related molecule. DDRs activate mechanisms to remove uncontrolled damaged cells and to repair DNA damage from apoptosis caused by chromatid instability [3]. Regulation of the DDR pathway is induced by post-translational modification (PTM); poly ADP-Ribosylation (PARylation) is the pivotal PTM that occurs rapidly at the damage site during DDR [4,5].

PARylation is the reaction of transferring ADP-ribose residues to target substrates by ADP-ribosyl transferase using NAD+. It rapidly recognizes multiple types of DNA damage, including SSBs, and is recruited to the damaged site to induce the recruitment of DDR molecules so that the poly (ADP-Ribose) polymerase (PARP), specifically PARP1, PARP2, PARP3, PARP5a, and PARP5b, which are known as the major molecules of DDR, performs poly (ADP-ribose) (PAR) synthesis in humans [6]. PARP1 was proposed as a new treatment for cancer, as the synthetic lethality concept suggested that its depletion in breast-cancer patients with germline mutations in the *BRCA1* or *BRCA2* genes, key molecules in the homologous recombination (HR) pathway, could cause cancer cell death [7,8]. Since it was proven to be true, PARP inhibitors that inhibit DDR resulted in improved clinical benefits and became standard therapy [9,10,11]. To date, four PARP inhibitors have been approved by the FDA and are being applied clinically. However, while all PARP inhibitors inhibit PARP catalytic activities, they have different cytotoxicities. Therefore, the anti-tumor effects of the PARP inhibitors have been suggested to be due to PARP trapping, as well as the inhibition of the enzymatic activities [12,13]. 

The catalytic inhibition and trapping effects of PARP are tightly regulated, and the cytotoxicity of each mechanism can cause different reactivities. Therefore, in this review, based on mechanisms of PARP, we intend to examine the difference of anti-tumor effect of the PARP inhibitors and the current aspect of the roles in combination treatment.

## 2. PARPs and PARylation

**Figure 1:**
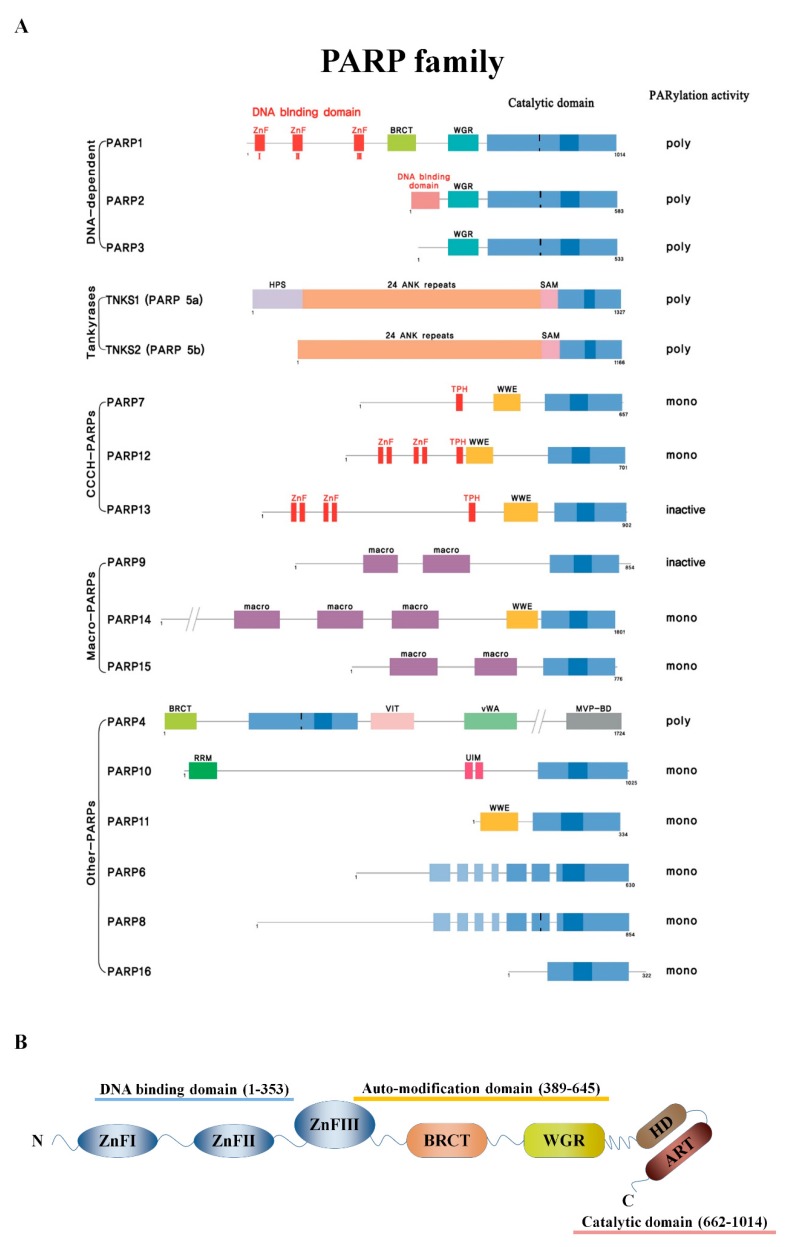
PARPs structure (**A**) The PARP family consists of 17 members, divided into five subgroups according to domain structure and function: DNA damage-dependent PARPs (PARP1, PARP2, and PARP3), tankyrases (tankyrase1/PARP5 and tankyrase2/PARP5b), CCCH-type PARPs (PARP7, PARP12, and PARP13), macro-PARPs [B-aggressive lymphoma 1 (BAL1)/PARP9, BAL2/PARP14, and BAL3/PARP15], and other PARPs (PARP4, PARP6, PARP8, PARP10, PARP11, and PARP16). The catalytic domain at the C-terminus is conserved in all members and contains additional zinc fingers, BRCA C-terminus-like (BRCT) motifs, ankyrin repeats, macro domains, and WWE domains. (**B**) The seven major domains of PARP1 include three zinc-finger domains in the DNA binding domain, the BRCT domain in the auto-modification domain, and the pADPr accepting WGR domain (W), located centrally. The C-terminus has two catalytic domains: ART and a helical domain (HD).

Poly (ADP-ribose) polymerase (PARP) is a family of 17 proteins in mammals, encoded by different genes, but with a conserved catalytic domain. Other than the catalytic domain, PARP family members contain one or more other motifs or domains, including zinc fingers, a breast cancer-susceptibility protein (BRCA) C-terminus-like (BRCT) motifs, ankyrin repeats, macro domains, and WWE domains [14] (Figure 1A). PARP1 was the first family member identified and has a critical role in SSB repair through the metabolism of recruiting and dissociating repair proteins by PARylation. In addition to DNA damage repair, PARP1 has important roles in a various range of cellular processes from cell proliferation to cell death, due to having diverse substrates like nuclear proteins involved in transcriptional regulation, apoptotic cell death, chromatin decondensation, inflammation, and cell cycle regulation [15,16]. PARP1 has a total molecular weight of 113 kDa and contains seven independent domains (Figure 1B) [5,17]. The N-terminus is the DNA binding domain (residues 1-353), which contains three zinc-finger DNA-binding domains, ZnFI, ZnFII, and ZnFIII, which are responsible for recognizing sites of damaged DNA and binding through allosteric activation. In the N-terminus there is a nuclear localization sequence (NLS) that places PARP1 in the nucleus with the KRK-X(11)-KKKSKK sequence. Between residues 211 and 214, there is a DEVD site that is cleaved by caspase into fragments of 23 and 89 KDa during apoptosis [18]. Residues 373 to 662 are the auto-modification domain consists of BRCA C-terminus-like (BRCT) domain serving sites of auto-ADP ribosylation and functioning in protein-protein interaction, and a WGR domain which roles in activating DNA damage repair by interaction with ZnFI, ZnFII, and catalytic domain. The auto-modification domain is rich in glutamate and lysine residues and is the site of self-PARylation. Finally, the C-terminus (residues 662–1014) is the catalytic domain, and the (ADP-Ribosyl) transferase (ART) domain is a NAD+ acceptor site where the His-Try-Glu residues called ART signatures are preserved well [19,20,21]. The helical subdomain (HD), an auto-inhibitory domain in the C-terminus, inhibits the binding of PARP1 and β-nicotinamide adenine dinucleotide without binding to DNA. When PARP1 binds to the DNA damage site, the auto-inhibitory function of HD is removed. The activation of the catalytic activity of ART and the generation of PAR chains in the target protein lead to the recruitment of DNA repair molecules. Thereafter, PARP1 is dissociated from DNA by auto-PARylation of PARP1, resulting in DNA repair [22].

This series of reactions is caused by PARylation. While the catalytic domain is conserved in the PARP family, only PARP1/2/3/4/5a/5b activates PARylation by possessing the His-Tyr-Glu motif called the “ART signature” [5,15,23,24,25,26]. The role of PARP3 as an (ADP-ribosyl) transferase is controversial. PARP4 is the largest protein in the PARP family, and PARP5a and PARP5b, classified as tankyrase1/2, have a SAM (Sterile Alpha motif) domain that interacts between proteins with the ability to homo- and hetero-oligomerize PARP1 and 2. PARP1 transfers ADP-ribose residues from NAD+ to acidic amino acid residues such as glutamates (E), lysine (K), arginine (R), serine (S), and aspartate (D), forming the negative poly (ADP-ribose) (PAR) chain [5,24,26]. PARP1 is believed to perform more than 90% of total PARylation in response to DNA damage. As soon as DNA damage occurs, ADP-ribosylation is covalently bound to the carbonyl group of the acidic residues of the target protein via ester bonds. PARP then forms a PAR chain by cleaving the glycosidic bond between nicotinamide and ribose of NAD+ by catalytic activity and binding ADP-ribosylation to the target protein via a 2′,1″-O-glycosidic bond [22,27]. PARylation in the DNA damage repair pathway plays a role throughout DNA strand breaks repair through rapid DNA repair molecules recruitment to the DNA damage site, DNA damage signal transduction, causing apoptosis and protein degradation. Typically, the BRCT domain of X-ray repair cross-complementing protein 1 (XRCC1) binds directly to the PAR chain to be recruited to the DNA damage site. Upon XRCC1 binding to DNA damage sites, the PAR formation is increased by sequestering poly (ADP-ribose) glycohydrolase (PARG) from the interaction with PARP1 and PARG, resulting in causing dissociation of PARP1 from DNA damage site and increasing repair signal transduction [26,27,28]. In addition, PARP1 has been reported to promote DNA repair by interacting with DNA glycosylase 8-oxoguanine glycosylase 1 (OGG1), XRCC1, DNA polymerase (DNAP) β, DNA ligase III, proliferating cell nuclear antigen (PCNA), aprataxin, and condensin I involved in BER and SSBR through PARylation of PARP1 [29,30].

## 3. Clinical Development of PARP Inhibitors

PARP inhibitors are nicotinamid analogs that inhibit PARylation through competitively binding to the NAD+ binding sites of PARP1 and PARP2. As a cancer treatment drug, olaparib is first defined as the HR deficient tumor treatment, and it is fully approved by FDA for serous ovarian cancer and breast cancer treatment with germline *BRCA1* or *BRCA2* mutations [9,31,32]. To date, four PARP inhibitors (olaparib, niraparib, rucaparib, and talazoparib) have been FDA approved, and veliparib is waiting for FDA approval with promising results of phase III trial showing significantly extended Progression-free survival (PFS) in combination with carboplatin plus paclitaxel in serous ovarian cancer, and the drugs are compared in Table 1.

It has been reported that the PARP inhibitor olaparib causes cell death by synthetic lethality in BRCA-deficient breast, ovarian, and prostate cancer. In a phase 2 trial, olaparib maintenance treatment in platinum-sensitive ovarian cancer patients improved progression-free survival (PFS) by 7 months [46]. In the SOLO-2 study, median PFS increased from 5.5 to 19.1 months over placebo in patients with BRCA1/2 mutations [11]. The OlympiAD trial, which was the global phase 3 trial for metastatic HER2-negative breast cancer with germline *BRCA1* or *BRCA2* mutations, confirmed that olaparib increased PFS by 2.8 months to 7.0 months (hazard ratio 0.58; P<0.001) and received FDA approval [9]. Not only for BRCA-deficient tumors, in the phase 2 STUDY-19 showed olaparib maintenance prolonged PFS in BRCA wild-type relapsed, platinum-sensitive serous ovarian cancer patients, extending the FDA approval to olaparib maintenance treatment in platinum-sensitive patients regardless of the BRCA status [47,48]. Moreover, PARP inhibitors were applied in prostate and pancreatic cancer, as well beyond the breast and ovarian cancers. The developed genomics technology verified that about 20 percent of prostate cancers have defects in DNA repair genes, resulting in a good candidate for PARP inhibitors [49]. In the phase II TOPARP-A trial, olaparib showed an 88 percent response rate in metastatic castrate-resistant prostate cancer (mCRPC) with BRCA1/2, ATM, or PALB2 mutation [50]. The PFS of patients with DNA repair gene defects increased from 2.7 to 9.8 months, and overall survival was also extended from 7.5 to 13.8 months. Based on these results, the FDA granted olaparib in breakthrough status in prostate cancer treatment, and several clinical trials are conducted in prostate cancer using PARP inhibitors. In addition, the median PFS was increased from 3.8 to 7.4 months with olaparib maintenance in the phase 3 POLO trial for metastatic pancreatic cancer patients with germline BRCA mutations who were sensitive to the first-line platinum-based therapy [37]. Recently, the FDA approved olaparib plus bevacizumab for the maintenance treatment of advanced ovarian cancer patients, who showed a response to the first-line platinum-based chemotherapy regardless of BRCA mutation. It is based on phase III PAOLA-1 trial results, in which the addition of olaparib to bevacizumab improved PFS significantly, to 37.2 months compared with 17.7 months in the placebo group among Homologous recombination deficiency (HRD)-positive ovarian cancer patients (HR, 0.33; 95% CI, 0.25–0.45) [51].

Rucaparib received FDA approval by demonstrating efficacy in a phase 2 trial of relapsed platinum-sensitive ovarian cancer patients who had previously received at least two platinum-based chemotherapies [38]. In addition, based on the phase 3 ARIEL3 trial, it received FDA approval for maintenance treatment of recurrent epithelial ovarian, fallopian tube, or primary peritoneal cancer with a partial or complete response to platinum-based chemotherapy [40]. 

In the case of niraparib, a randomized and double-blind phase 3 trial in 553 platinum-sensitive, recurrent ovarian cancer patients showed an increase in PFS from 5.5 to 21.0 months relative to placebo in the presence of germline BRCA mutations [41]. The FDA approval was granted based on the phase 3 trial on patients with recurrent epithelial ovarian, fallopian tube, or primary peritoneal cancer who were in complete or partial response to platinum-based chemotherapy [41]. 

Talazoparib has been FDA-approved for its efficacy against cancers with germline *BRCA1* or *BRCA2* mutations and HER2-negative metastatic breast cancer [52]. In the phase 3 EMBRACA trial, upon which FDA approval was based, talazoparib significantly increased median PFS from 5.6 to 8.6 months compared to physician’s choice standard-of-care chemotherapy. The objective response rate was more than doubled over that of the control arm (62.6% for talazoparib vs. 27.2% for chemotherapy [OR: 4.99 (95% CI: 2.9–8.8), *p* < 0.0001]) [53]. 

Veliparib is another potent inhibitor of PARP1 and PARP2 in the developmental stage. In the phase III VELIA trial, which involved adding veliparib to first-line induction chemotherapy with carboplatin and paclitaxel followed by maintenance monotherapy in serous ovarian cancer increased median PFS from 17.3 to 23.5 months regardless of BRCA or HRD status [54]. Besides, veliparib 120mg bid plus carboplatin AUC6 and paclitaxel 200 mg/m^2^ treatment showed improved PFS from 4.2 to 5.8 months and OS from 8.4 to 10.3 months in phase II trial of Non-small-cell lung cancer (NSCLC) [55]. These results contributed that FDA grants orphan drug designation to veliparib for advanced NSCLC. Veliparib, in addition to carboplatin and paclitaxel, also significantly improved PFS to patients with HER2-negative advanced or metastatic breast cancer with germline BRCA-mutation in a phase III trial. The rate of 3-year PFS was 26 percent on veliparib addition group when the placebo group showed 11 percent [45]. Although veliparib is not yet approved to FDA on any indications, these data could accelerate the application of veliparib in the clinics. 

PARP inhibitors in the clinic showed improved clinical benefits, not only for tumors with *BRCA* mutations, but also for platinum-sensitive tumors caused by HRD, leading to their accelerated clinical application. However, the effects of these PARP inhibitors are difficult to understand as an inhibition of catalytic activity that simply inhibits PARylation. Although all four PARP inhibitors can inhibit the catalysis by PARP1 and PARP2, as shown in Table 1, each PARP inhibitor shows different cytotoxicity. PARP inhibition by PARP inhibitors induces a cytotoxicity far superior to the cytotoxicity induced by the knockout of PARP genes, suggesting that their antitumor effects are due to mechanisms other than the catalytic inhibition of PARP [12,13,34,56]. 

This difference can be conceptualized as PARP trapping: the ability of PARP inhibitors to trap PARP-DNA complexes while increasing the stability of the binding between PARP and DNA. As shown in Table 1, each PARP inhibitor has a different cytotoxicity that correlates with its PARP trapping activity. In other words, talazoparib, with the strongest PARP trapping effect, also is the most cytotoxic. Therefore, PARP trapping should be considered as a mechanism for the application of PARP inhibitors in clinical trials [34,57,58]. These differences in PARP trapping capacity may have different effects on combination therapy, as well as on monotherapy. Indeed, reactivities differ with different combination partners for each drug. 

## 4. Combination Effect of Conventional Chemotherapy according to the Mechanism of Action of PARP Inhibitors

**Figure 2:**
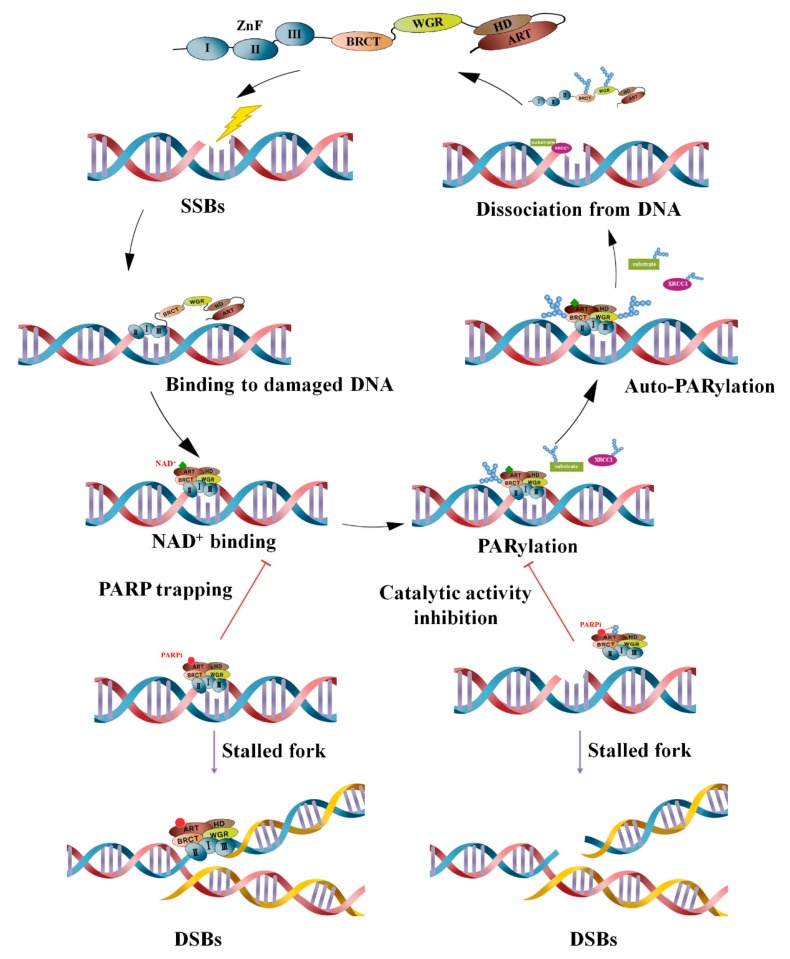
Mechanisms of PARylation and PARP inhibition in the DNA damage response. When a DNA single-strand break occurs, PARP quickly binds to the damage site using a zinc finger domain. It causes recruitment of DNA repair proteins to DNA damage sites by catalyzing PARylation between PARP and its target proteins XRCC1, DNA ligase III, etc., by the ART catalytic domain using NAD+ as a substrate. PARP auto-PARylation then decreases the affinity for DNA, resulting in dissociation from DNA so that the repair protein can bind. At this time, the PARP inhibitor binds to the pocket instead of NAD+, causing PARP to be trapped in the DNA. It encounters a replication fork, causing it to stall, and is converted into double-strand breaks (DSBs) leading to cell death; alternatively, it blocks the recruitment of repair proteins by blocking enzymatic activity where PARP PARylation occurs.

PARP inhibitors inhibit the catalytic activity of NAD+ depletion through competitive binding with NAD+, thereby inhibiting PARP itself as well as PARylation of target proteins in the nucleus. Not only do they cause cytotoxicity via irreparable damage caused by inhibiting repair protein recruitment to DNA damage sites, but also, they block cellular replication by inducing stalling or collapsing of the replication fork, as the PARP protein is continuously trapped in SSBs due to suppressed dissociation of PARP from DNA. This results in more deleterious DSBs, which in turn leads to cell death (Figure 2) [59,60,61].

PARP inhibitors were originally developed to sensitize tumors to the effects of DNA damaging agents, including ionizing radiation, temozolomide, and topotecan. In fact, PARP inhibitors have successfully sensitized tumors to radiation and to camptothecin, a topoisomerase I inhibitor [62,63]. However, the combination of gemcitabine, doxorubicin, and taxan showed no significant synergies [64,65]. It was observed that the efficacy of combination therapy with the same chemoagent was dependent on PARP inhibitors [66]. PARP inhibitors are thought to have different synergies in combinations with different chemoagents, depending on the mechanism and activity of cytotoxicity, as well as the regulation of PARylation. The combined effect of PARP inhibitors with different classes of chemoagents in the clinic is summarized in Table 2, in accordance with the mechanism of action of PARP inhibitors.

**Figure 3:**
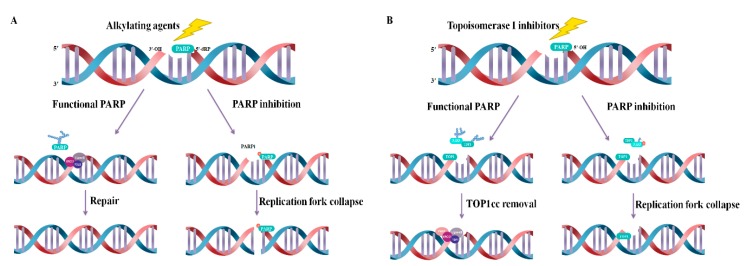
Principle of combination therapy with chemoagents, based on trapping effects and inhibition of catalytic activity. (**A**) An alkylating agent forms a single-nucleotide gap with 5’-deoxyribose phosphate (5-dRP). PARP1/2 senses and binds it, inducing recruitment of BER molecules. A PARP inhibitor prevents repair by inhibiting dissociation of PARP via trapping the PARP-DNA complex, which is PARP bound to 5-dRP. (**B**) TOP1cc, a covalent binding state of TOP1 induced by Top1 and a DNA 3′-end, is repaired by TDP1 recruited to TOP1cc by PARylation and PAR transferase of PARP1 to induce TOP1-DNA complex excision. Topoisomerase I inhibitors continuously induce TOP1cc. PARP inhibitors inhibit catalytic activity, suppressing the recruitment of TDP1 and TOP1-DNA covalent complex repair, resulting in a synergistic effect.

Their synergistic effect on chemoagents by inhibition of catalytic activity is well defined by topoisomerase I inhibitors, topotecan and camptothecin. The synergistic effect of combination therapy with alkylating agents such as MMS is well defined by the PARP-DNA trapping activity of PARP inhibitors (Figure 3) [77,78,79]. First, alkylating agents generate basic sites that consist of a 1-nucleotide gap with 3′-OH and 5′-deoxyribose phosphate (5′-dRP) groups at the ends of the breaks created by APEX1 endonuclease. PARP1 binds directly to 5’-dRP and recruits BER proteins to induce repair. However, PARP inhibitors bind PARP to the 5-dRP end, trapping and maintaining the PARP-DNA complex so that the accumulation of SSBs leads to DSBs, and ultimately, cell death [60,61,66,78,80]. This synergistic effect based on PARP trapping is the strongest for talazoparib. On the other hand, a topoisomerase I inhibitor causes SSBs by the endonuclease activity of TOP1 and causes DNA damage by trapping TOP1cc covalently bonded with TOP1 at the DNA 3’ end. At this time, the PARP inhibitor sustains the trapping of TOP1cc by inhibiting PARP1 recruitment of TDP1 through PARylation, so that TDP1 can remove the covalent attachment of TOP1 by phosphodiesterase activity. At this point, the PARP inhibitor lets the trapping of TOP1cc continue by preventing TDP1 from removing the covalent attachment of TOP1, as PARP1 recruits TDP1 through PARylation. This is due to the regulation of PARylation activity by PARP inhibitors, which has the same effect on all PARP inhibitors developed in the clinic [63,77,79]. Those applying combination therapies of PARP inhibitors and chemotherapy within the clinic should carefully consider the mechanism(s) of action prior to selecting the drug.

## 5. Conclusions

PARP performs a variety of functions from the transcriptional level, to activation and localization through post-translational modification. In the DNA damage response, PARP contributes to the activation of itself or its target protein through the regulation of PARylation. PARP inhibitors block this catalytic activity of PARP, preventing the activation of normal repair pathways. These PARP inhibitors have demonstrated dramatic anti-tumor effects for tumors with HRD, such as those with BRCA mutations. To date, four PARP inhibitors have been approved by the FDA and applied in clinical practice. However, these four have different effects on the trapping of the PARP-DNA complex, despite inhibiting the common catalytic activity of PARP. This PARP trapping leads to improved cytotoxicity via replication fork collapse, leading to conversion to DSBs. When PARP inhibitors are used in combination with alkylating agents, synergistic effects are achieved. In contrast, inhibition of the catalytic activity of PARP has a synergistic effect when combined with topoisomerase I inhibitors. In other words, the synergies of combination therapies with PARP inhibitors can be induced differently depending on the mechanism of action of individual PARP inhibitors. Understanding the characteristics of each PARP inhibitor to strategically select synergistic partners is an important matter that must be considered to produce maximum antitumor effects.

## Figures and Tables

**Table 1:** Comparison of available PARP inhibitors in the clinics.

Agents	Company	Target	Application in Clinics			Mean Half-Life (Hours)	Catalytic Inhibition (IC_50_ in Wild-Type DT40 Cells; nM) [13]	PARP Trapping Potency (Relative to Olaparib) [33]	Cytotoxicity (EC_50_ in BRCA2 Mutated Capan-1 Cells; nM) [34]
			Indication	Clinical Trials Based on FDA Approval	Dosage				
**Olaparib**	AstraZeneca	PARP1 PARP2 PARP3	Maintenance treatment of germline BRCA-mutated advanced ovarian cancer.	**Study 42** (NCT01078662) [35]	300mg BID	14.9 ± 8.2	6	1	259
			Maintenance treatment of recurrent serous ovarian cancer regardless of BRCA mutations	**SOLO-2** (NCT01874353) [11] **Study 19** (NCT00753545) [36]					
			Treatment of germline BRCA-mutated HER2-negative locally advanced or metastatic breast cancers	**OlympiAD** (NCT02000622) [9]					
			First-line maintenance treatment of germline BRCA-mutated metastatic pancreatic cancer	**POLO trial** (NCT02184195) [37]					
**Rucaparib**	Clovis Oncology	PARP1 PARP2 PARP3	Treatment of germline and/or somatic BRCA-mutated advanced ovarian cancer	**ARIEL2** (NCT01891344) [38] **Study 10** (NCT01482715) [39]	600mg BID	18 ± 1	21	1	609
			Maintenance treatment in a platinum-sensitive recurrent epithelial ovarian, fallopian tube, or primary peritoneal cancer	**ARIEL3** (NCT01968213) [40]					
**Niraparib (MK4827)**	Tesaro	PARP1 PARP2	Maintenance treatment of platinum-sensitive, recurrent ovarian cancer	**ENGOT-OV16/NOVA** (NCT01847274) [41]	300mg QD	36	60	2	650
			Treatment of homologous recombination deficiency (HRD) positive advanced ovarian, fallopian tube, or primary peritoneal cancer	**QUADRA** (NCT02354586) [42]					
**Talazoparib (BMN-673)**	Pfizer	PARP1 PARP2	Treatment of germline BRCA-mutated HER2-negative locally advanced or metastatic breast cancers	**EMBRACA** (NCT01945775) [43]	1mg QD	90	4	100	5
**Veliparib** **(ABT-888)**	Abbott Laboratories	PARP1 PARP2	Not yet approved for any indication, but in 2014. FDA awards orphan drug designation to Veliparib for advanced non-small cell lung cancer (NSCLC). In phase III trial, veliparib significantly improved progression-free survival in BRCA-mutated or HRD cohort compared to carboplatin plus paclitaxel (NCT02470585) [44]. In phase III trial of veliparib with carboplatin and paclitaxel in advanced HER2-negative breast cancer with germline BRCA mutation, median PFS in patients treated with veliparib plus carboplatin and paclitaxel was 14.5 months compared to 12.6 months in placebo plus carboplatin and paclitaxel (BROCADE3; NCT02163694) [45].			5.2	30	<0.2	>10,000

**Table 2:** Combination effects of PARP inhibitors and chemotherapeutics according to the action mechanism of PARP inhibitors on the combination strategies.

Action Mechanism of PARP Inhibitors on the Combination Effects	Combined Chemotherapeutics		PARP Inhibitor	Tumor Type	Trial	Phase	Outcome
	Class of Agents	Chemotherapy Agents					
**Inhibition of PARP catalytic activity**	Topoisomerase I inhibitors	Topotecan	Olaparib	Advanced solid tumors	NCT00516438 [67]	I	The Maximum Tolerated Dose (MTD) was determined as topotecan 1.0 mg/m^2^/day × 3 days plus olaparib 100 mg bid.
			Veliparib	Recurrent cervix cancer	NCT01266447 [68]	II	Topotecan 0.6 mg/m^2^/day × 5 days plus veliparib 10 mg bid treatment resulted in 7% partial response (PR).
	Platinum-based inhibitors	Carboplatin	Olaparib	Refractory or recurrent breast and ovarian cancer	NCT01237067 [69]	I	The MTD as olaparib 200 mg bid plus carboplatin AUC4; The responses including CRs and PRs was higher in BRCA mutation carriers compared with nonmutation carriers (68% vs 19%)
			Veliparib	HER2-negative metastatic breast cancer	NCT01251874 [70]	I	The MTD was established as veliparib 250 mg bid plus carboplatin AUC5.
			Rucaparib	Advanced solid tumors	NCT01009190 [71]	I	The MTD for combination was established as 240 mg/day oral rucaparib and carboplatin AUC5.
**PARP trapping**	Alkylating agents	Temozolomide	Olaparib	Relapsed glioblastoma	NCT01390571 [72]	I	The temozolomide 75 mg/m^2^ daily plus olaparib 150 mg/day × 21 days treatment was well tolerated and encouraged 6 months progression-free survival rates.
			Veliparib	Metastatic breast cancer and BRCA1/2 mutated breast cancer	NCT01506609 [73]	II	The responses in the BRCA mutation carriers showed a total response rate (RR) 25% (7/28) and clinical benefit rate (CBR) 50%.
		Mitomycin C	Veliparib	Metastatic, unresectable or recurrent solid tumors	NCT01017640 [74]	I	Veliparib 200 mg bid after treated mitomycin C 10 mg/m^2^/day × 21 days was recommended for combination treatment.
**Inhibition of PARP transcription cofactor function**	Antimetabolite	Gemcitabine	Olaparib	Pancreatic cancer	NCT00515866 [75]	I	Olaparib 100 mg bid plus gemcitabine 600 mg/m^2^/week was tolerated and recommended for the phase II trial.
**Unknown mechanism**	taxanes	paclitaxel	Olaparib	Metastatic triple-negative breast cancer (TNBC)	NCT00707707 [64]	I	Olaparib 200 mg bid daily in combination with paclitaxel 90 mg/m^2^/week × 3 of 4 weeks was tolerated; The overall response rate (ORR) was 33.3%.
				Advanced gastric cancer	NCT01063517 [76]	II	Olaparib 100 mg bid plus paclitaxel 80 mg/m^2^ treatment showed overall survival and progression-free survival benefit in ATM enriched phase II study.
					NCT01924533 [32]	III	In phase III study, median overall survival was 8.8 months in the weekly paclitaxel 80 mg/m^2^ + olaparib 100 mg bid vs 6.9 months in the paclitaxel + placebo group; HR 0.79, *p* = 0.026, without statistical significance.

The table is based on the details from http://www.clinicaltrials.gov/.
